# Supplementary material for: Modelling Skylarks (Alauda arvensis) to Predict Impacts of Changes in Land Management and Policy: Development and Testing of an Agent-Based Model
Source: PLoS One. 2013 Jun 6;8(6):e65803. doi: 10.1371/journal.pone.0065803 (PMC3675089; doi:10.1371/journal.pone.0065803)
Supplement: Supporting Information S4 — The skylark ODdox as a zipped archive. (ZIP) [file pone.0065803.s004.zip › Skylark_ODdox/weather_8h.html]

ALMaSS Skylark ODdox: weather.h File Reference


ALMaSS Skylark ODdox
 2.0


- Main Page
- Related Pages
- Classes
- Files

- File List
- File Members

Classes |
Variables

weather.h File Reference

`#include <vector>`

|  |  |
| --- | --- |
| Classes | |
| class | Weather |

|  |  |
| --- | --- |
| Variables | |
| const double | c\_insolation [365] |
| class Weather \* | g\_weather |

---

## Variable Documentation

const double c\_insolation[365]

Referenced by Weather::GetGlobalRadiation(), and Weather::Tick().

class Weather\* g\_weather

Referenced by Farm::AutumnHarrow(), Farm::AutumnPlough(), Farm::AutumnRoll(), Farm::AutumnSow(), Farm::BurnStrawStubble(), Farm::CutToHay(), Farm::CutToSilage(), Farm::CutWeeds(), Farm::DeepPlough(), VegElement::DoDevelopment(), UnsprayedFieldMargin::DoDevelopment(), Skylark\_Population\_Manager::DoFirst(), Farm::FA\_AmmoniumSulphate(), Farm::FA\_GreenManure(), Farm::FA\_Manure(), Farm::FA\_NPK(), Farm::FA\_PK(), Farm::FA\_Sludge(), Farm::FA\_Slurry(), Farm::FP\_GreenManure(), Farm::FP\_LiquidNH3(), Farm::FP\_ManganeseSulphate(), Farm::FP\_Manure(), Farm::FP\_NPK(), Farm::FP\_NPKS(), Farm::FP\_PK(), Farm::FP\_Sludge(), Farm::FP\_Slurry(), Farm::FungicideTreat(), Farm::GrowthRegulator(), Farm::Harvest(), Farm::HayBailing(), Farm::HayTurning(), Farm::HerbicideTreat(), Farm::HillingUp(), Farm::InsecticideTreat(), Landscape::Landscape(), Farm::Molluscicide(), Farm::ProductApplication0(), Farm::ProductApplication1(), Farm::RowCultivation(), VegElement::SetGrowthPhase(), Farm::SpringHarrow(), Farm::SpringPlough(), Farm::SpringRoll(), Farm::SpringSow(), Farm::StrawChopping(), Farm::Strigling(), Farm::StriglingSow(), Farm::StubbleHarrowing(), Landscape::SupplyGlobalRadiation(), Landscape::SupplyMeanTemp(), Landscape::SupplyRain(), Landscape::SupplyRainPeriod(), Landscape::SupplySnowcover(), Landscape::SupplyTemp(), Landscape::SupplyTempPeriod(), Landscape::SupplyWind(), Landscape::SupplyWindPeriod(), Farm::Swathing(), Farm::SynInsecticideTreat(), Landscape::Tick(), Farm::Water(), Farm::WinterPlough(), and Landscape::~Landscape().


- CJT
- MSVC
- ALMaSS Working Source
- Landscape
- weather.h
- Generated on Thu Jan 10 2013 13:15:35 for ALMaSS Skylark ODdox by
   1.8.1.1
